# Promoting crystallization of antibody–antigen complexes *via* microseed matrix screening

**DOI:** 10.1107/S0907444910026041

**Published:** 2010-07-14

**Authors:** Galina Obmolova, Thomas J. Malia, Alexey Teplyakov, Raymond Sweet, Gary L. Gilliland

**Affiliations:** a Centocor R&D, 145 King of Prussia Road, Radnor, PA 19087, USA

**Keywords:** crystallization, microseed matrix screening, antibody–antigen complexes

## Abstract

The application of microseed matrix screening to the crystallization of antibody–antigen complexes is described for a set of antibodies that include mouse anti-IL-13 antibody C836, its humanized version H2L6 and an affinity-matured variant of H2L6, M1295. The Fab fragments of these antibodies were crystallized in complex with the antigen human IL-13. The initial crystallization screening for each of the three complexes included 192 conditions. Only one hit was observed for H2L6 and none were observed for the other two complexes. Matrix self-microseeding using these microcrystals yielded multiple hits under various conditions that were further optimized to grow diffraction-quality H2L6 crystals. The same H2L6 seeds were also successfully used to promote crystallization of the other two complexes. The M1295 crystals appeared to be isomorphous to those of H2L6, whereas the C836 crystals were in a different crystal form. These results are consistent with the concept that the conditions that are best for crystal growth may be different from those that favor nucleation. Microseed matrix screening using either a self-seeding or cross-seeding approach proved to be a fast, robust and reliable method not only for the refinement of crystallization conditions but also to promote crystal nucleation and increase the hit rate.

## Abstract 2

The application of microseed matrix screening to the crystallization of related antibodies in complex with IL-13 is described. Both self-seeding or cross-seeding helped promote nucleation and increase the hit rate.

## 1. Introduction

Information on the three-dimensional structure of antibody–antigen complexes is essential for antibody engineering and for understanding their mechanism of action. It is recognized that X-ray crystallography provides the most accurate and detailed data on protein conformation and interactions. This method depends on the production of well ordered single crystals of the macromolecule or complex of interest that diffract X-rays.

The crystallization of macromolecules has advanced in recent years with the use of protein engineering to enhance the crystallizability of proteins (Derewenda, 2004 ▶, 2010 ▶; Bolanos-Garcia & Chayen, 2009 ▶), the application of fast screens (Jancarik & Kim, 1991 ▶; Stura *et al.*, 1992 ▶; Riès-Kautt & Ducruix, 1997 ▶; Brzozowski & Walton, 2001 ▶; McPherson & Cudney, 2006 ▶) and of the use of robotics, which allows the screening of a large number of crystallization conditions in a miniaturized format, reducing the amount of protein needed (Stevens, 2000 ▶; Snook *et al.*, 2000 ▶; Weselak *et al.*, 2003 ▶; Rupp, 2003 ▶). Another major development that advanced the field was the application of various microseeding techniques for crystal optimization (Stura, 1999 ▶; Bergfors, 2003 ▶). Seeding exploits the hypothesis that the optimal conditions needed for crystal nucleation and for crystal growth can be quite different (Kam *et al.*, 1978 ▶). Traditionally, two general approaches, microseeding and macroseeding, have been used to produce single crystals of macromolecules (Bergfors, 2003 ▶). These approaches typically use microseeds or macroseeds produced from the macromolecule of interest. In some cases, seeding with the crystals of a sequence variant or homologous protein has proven to be successful (Stura & Wilson, 1991 ▶; Walter *et al.*, 2008 ▶). This cross-seeding approach can be used for related proteins that may include complexes with various ligands, heavy-atom derivatives and homologous proteins such as the Fab fragments of monoclonal antibodies (mAbs).

The use of seeding has been extended by the ‘microseed matrix screening’ (MMS) approach, in which seeds are systematically transferred into new conditions to promote crystal growth (Ireton & Stoddard, 2004 ▶). The use of MMS has become an essential part of the screening process in several laboratories employing self-seeding (D’Arcy *et al.*, 2007 ▶) or cross-seeding with protein derivatives (Walter *et al.*, 2008 ▶). Recently, a study investigating this methodology provided evidence that in some cases the seed-stabilization solution by itself can induce crystallization as effectively as the presence of seeds (St John *et al.*, 2008 ▶). Regardless of the underlying mechanism, the MMS technique has great potential for improving hit rates in the screening for crystallization conditions.

We have applied the MMS method to the crystallization of antibody–antigen complexes and report here the successful crystallization of three IL-13 complexes with different but related Fab fragments: C836, H2L6 and M1295. C836 is a mouse hybridoma mAb against human IL-13. The C836 mAb binds IL-13 with high affinity and blocks the binding of IL-13 to its receptors. The variable (V) regions of this mAb were chimerized to human G1 and kappa constant regions. This antibody was further humanized by grafting the complementarity-determining regions (CDRs) into human variable framework segments to yield H2L6 mAb. H2L6 was subsequently affinity-matured by selection of CDR variants to produce M1295 mAb (Fransson *et al.*, 2010 ▶). In terms of the amino-acid sequence, H2L6 differs from C836 at 40 positions in the 228 residues of the variable domains. M1295 differs from H2L6 at only four positions. All three Fabs contained the same human constant domains. The crystals of the complexes were used in their structure determinations, which have been reported previously (Fransson *et al.*, 2010 ▶; the atomic models were deposited in the PDB under accession codes 3l5w, 3l5x and 3l5y).

In this paper, we describe a crystallization routine that includes the following steps: (i) conventional ‘fast screening’ with commercial kits, (ii) selection of hits and preparation of the seed stock, (iii) MMS in a subset of the initial screen and (iv) final optimization of conditions if needed. Both self-seeding and cross-seeding proved to be effective in producing diffraction-quality crystals. Application of the MMS method increased the hit rate and consequently reduced the number of experiments and the amount of protein needed.

## 2. Materials and methods

### 2.1. Proteins

Recombinant human IL-13 was purchased from R&D Systems (Minneapolis, Minnesota, USA; catalog No. 213-IL/CF). The protein was reconstituted in phosphate-buffered saline (PBS) pH 7.4 according to the manufacturer’s protocol.

His-tagged C836 Fab was expressed in CHO cells. The H2L6 and M1295 Fabs were prepared by papain cleavage of the corresponding mAbs. All Fab proteins were purified using affinity and size-exclusion chromatography as described previously (Fransson *et al.*, 2010 ▶).

### 2.2. Complex preparation

For complex formation, purified C836, H2L6 and M1295 Fab fragments were buffer-exchanged into 20 m*M* Tris–HCl pH 7.4, 50 m*M* NaCl. The complexes were prepared by mixing each Fab with IL-13 at a Fab:IL-13 molar ratio of 1:1.2 (excess IL-13). The mixture was incubated for 20 min at 277 K, con­centrated to a final volume of 0.6 ml using an Amicon Ultra 5 kDa device (Millipore) and loaded onto a Superdex 200 10/300 column (GE Healthcare, Piscataway, New Jersey, USA) equilibrated with 20 m*M* HEPES pH 7.5, 0.1 *M* NaCl. A shift in the elution profile (elution earlier than the free Fab) indicated complex formation. Three runs were performed, with 0.2 ml protein solution applied each time to the column for each complex. Fractions corresponding to the main peak were pooled, concentrated to 6–9 mg ml^−1^ in 20 m*M* HEPES pH 7.5, 0.1 *M* NaCl and used in crystallization trials.

### 2.3. Crystallization screening

Crystallization of the complexes was carried out by the sitting-drop vapor-diffusion method at 293 K. Screening for crystallization conditions was carried out using a Hydra II eDrop robot (Thermo Scientific, Waltham, Massachusetts, USA) to set up crystallization trials in 96-well Corning 3550 plates (Corning, New York, USA). The experiments were composed of 0.5 µl protein solution mixed with an equal volume of reservoir solution. The droplets were equilibrated against 90 µl reservoir solution. Optimization screens were made using a Matrix Maker (Emerald BioSystems, Bainbridge Island, Washington, USA).

### 2.4. Seed-stock preparation and microseed matrix screening

Microcrystals used for seed-stock preparation were placed in 100 µl reservoir solution, homogenized by vortexing for 3 min with a Teflon Seed Bead (Hampton Research, Aliso Viejo, California, USA) and stored at 253 K. The MMS was set up manually using the hanging-drop vapor-diffusion method in 24-well VDX greased plates (Hampton Research, Aliso Viejo, California, USA). In each crystallization drop, 0.6 µl screening (reservoir) solution and 0.2 µl microseeds were added to 0.8 µl protein solution. The protein droplets were equilibrated over 500 µl reservoir solution.

## 3. Results

### 3.1. Initial crystallization screening

**Figure 1:**
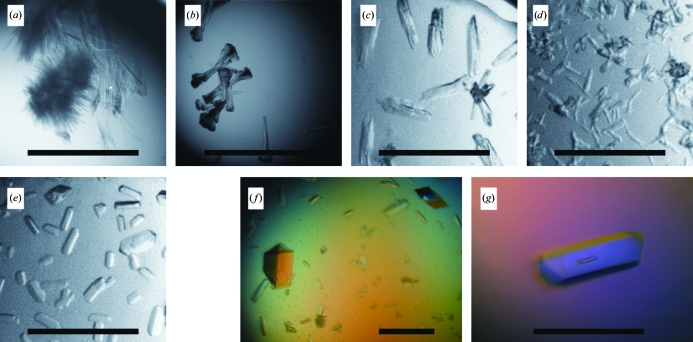
(*a*) Microcrystals of H2L6 (28% PEG 8000, 0.1 *M* MES pH 6.5) used for MMS. (*b*–*e*) H2L6 crystals after the first round of MMS obtained under the following conditions: (*b*) 20% PEG 3350, 0.2 *M* lithium acetate pH 7.9, (*c*) 20% PEG 3350, 0.2 *M* ammonium tartrate pH 6.6, (*d*) 20% PEG 3350, 0.2 *M* ammonium phosphate pH 8.0, (*e*) 20% PEG 3350, 0.2 *M* ammonium citrate pH 5.1. (*f*, *g*) Diffraction-quality H2L6 crystals after the second round of MMS obtained under the following conditions: (*f*) 14% PEG 3350, 0.2 *M* ammonium tartrate, 0.1 *M* MES pH 6.5, (*g*) 16% PEG 3350, 0.2 *M* ammonium citrate, 0.1 *M* MES pH 6.5. Scale bars are 0.3 mm in length.

The initial screening was performed with Crystal Screens I and II, PEG/Ion Screen (Hampton Research, Aliso Viejo, California, USA) and in-house grid screens: 192 conditions in total. The in-house screens, PEG 8000/pH and ammonium sulfate/pH, each containing 24 conditions, were designed in a small 6 × 4 matrix format. In these screens the concentration of the precipitating agent varied from 18 to 34% for PEG 8000 (all PEG concentrations in this paper are given as weight/volume percentage solutions) and from 1.5 to 2.4 *M* for ammonium sulfate *versus* a pH range of 3.5–10.5. Needle-like microcrystals of the H2L6 complex were observed in 28% PEG 8000, 0.1 *M* MES pH 6.5 (Fig. 1 ▶ *a*). The other two com­plexes did not produce any hits. Optimization of the H2L6 complex crystallization conditions in a standard approach of refining the PEG 8000 concentration and using various additives did not improve the original needle-like crystals. Therefore, the H2L6 complex microcrystals were used as seeds in the second screening by the microseed matrix method (Ireton & Stoddard, 2004 ▶; D’Arcy *et al.*, 2007 ▶) for all three complexes.

### 3.2. H2L6 complex

MMS was performed with the Hampton Research PEG/Ion Screen (48 conditions). This screen was extended by the addition of eight conditions containing 14–22% PEG 8000 or 1.6–2.4 *M* ammonium sulfate both in 0.1 *M* MES pH 6.5, representing an optimization screen for the H2L6 complex microcrystals.

Small isometric crystals were observed after 24 h from PEG/Ion Screen under several conditions, all of which contained 20% PEG 3350 plus one of the following salts: 0.2 *M* lithium acetate pH 7.9 (condition No. 24), 0.2 *M* ammonium tartrate pH 6.6 (No. 38), 0.2 *M* ammonium phosphate pH 8.0 (No. 44) or 0.2 *M* ammonium citrate pH 5.1 (No. 48) (Figs. 1 ▶ *b*–1 ▶ *e*). No crystals were observed in the experiments using the eight additional conditions.

The new crystallization hits were optimized using a screen composed of the most promising salt/PEG 3350 combinations (24 conditions). The second MMS was performed with the same seeds, but the seed stock was diluted fivefold with 30% PEG 8000, MES pH 6.5 to minimize nucleation events.

X-ray-quality crystals were obtained from 14% PEG 3350, 0.2 *M* ammonium tartrate, 0.1 *M* MES pH 6.5 and from 16% PEG 3350, 0.2 *M* ammonium citrate, 0.1 *M* MES pH 6.5. The crystals appeared within 2 d and reached dimensions of 0.1 × 0.1 × 0.3 mm (Figs. 1 ▶ *f* and 1* ▶g*). Both conditions produced the same crystal form. The crystals from ammonium tartrate diffracted to 1.9 Å resolution and were used for structure determination. They belonged to the orthorhombic space group *P*2_1_2_1_2_1_, with unit-cell parameters *a* = 63.78, *b* = 73.02, *c* = 114.86 Å. The asymmetric unit contained one molecule of the complex.

### 3.3. M1295 complex

**Figure 2:**
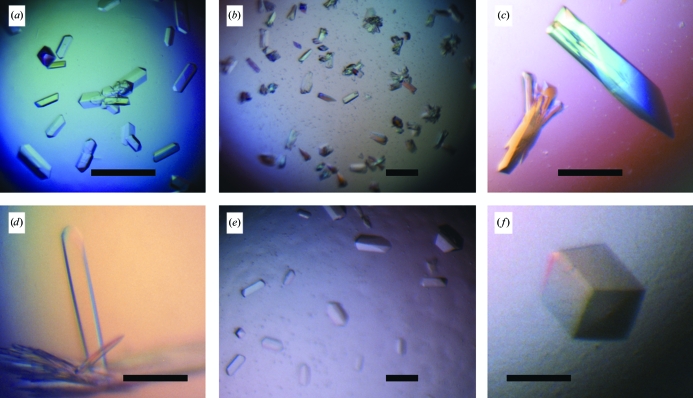
(*a*) Crystals of H2L6 (20% PEG 3350, 0.2 *M* ammonium citrate pH 5.1) used to generate a seed stock for MMS crystallization of M1295 and C836. (*b*–*f*) Crystals of M1295 obtained by MMS under the following conditions: (*b*) 20% PEG 3350, 0.2 *M* lithium chloride pH 6.8, (*c*) 20% PEG 3350, 0.2 *M* potassium chloride pH 7.0, (*d*) 20% PEG 3350, 0.2 *M* sodium citrate pH 8.3, (*e*) 25% PEG 8000, 0.1 *M* sodium acetate pH 5.5, (*f*) 25% PEG 8000, 0.1 *M* sodium acetate pH 4.5. Scale bars are 0.2 mm in length.

The optimized H2L6 crystals obtained from 16% PEG 3350, 0.2 *M* ammonium citrate, 0.1 *M* MES pH 6.5 (Fig. 2 ▶ *a*) were used to prepare seeds for the M1295 complex crystallization. The initial MMS included the 192 crystallization conditions described above. Crystals were obtained directly from this screen in the following conditions from PEG/Ion Screen: 20% PEG 3350, 0.2 *M* lithium chloride pH 6.8 (condition No. 4; Fig. 2 ▶ *b*), 20% PEG 3350, 0.2 *M* potassium chloride pH 7.0 (No. 8; Fig. 2 ▶ *c*) and 20% PEG 3350, 0.2 *M* sodium citrate pH 8.3 (No. 46; Fig. 2 ▶ *d*). In addition, X-ray-quality crystals grew from 25% PEG 8000, 0.1 *M* sodium acetate pH 5.5 (in-house grid screen; Fig. 2 ▶ *e*). The latter conditions were optimized (pH 4.5) to yield crystals of about 0.2 × 0.2 × 0.2 mm in 2 d (Fig. 2 ▶ *f*). These crystals diffracted to 2.8 Å resolution and were used for structure determination. The crystals have the same space group and nearly identical unit-cell parameters as the H2L6 crystals that were used as seeds. The space group is *P*2_1_2_1_2_1_, with unit-cell parameters *a* = 63.37, *b* = 72.50, *c* = 114.20 Å. The asymmetric unit contains one molecule of the complex.

### 3.4. C836 complex

**Figure 3:**
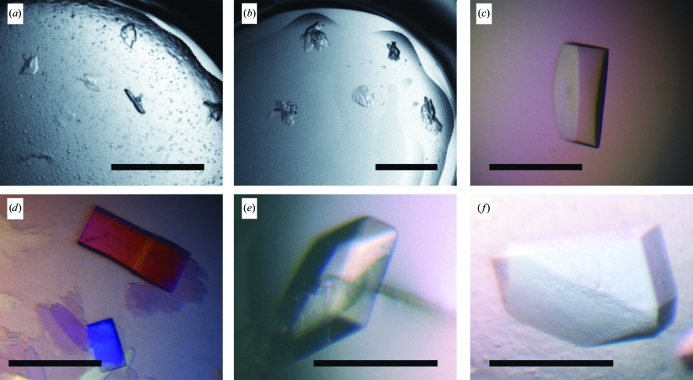
Crystals of C836. (*a*, *b*) Microcrystals used to generate a self-seed stock obtained from the following conditions: (*a*) 25% PEG 8000, 0.1 *M* HEPES pH 7.5, (*b*) 20% PEG 3350, 0.2 *M* lithium citrate pH 8.4. (*c*–*f*) Diffraction-quality crystals of C836 obtained by MMS under the following conditions (all contain 0.1 *M* HEPES pH 7.5): (*c*) 18% PEG 3350, 0.2 *M* ammonium citrate, self-seeds, (*d*) 20% PEG 3350, 0.2 *M* ammonium citrate, cross-seeds, (*e*) 22% PEG 3350, sodium formate, cross-seeds, (*f*) 20% PEG 3350, 0.2 *M* sodium tartrate, cross-seeds. Scale bars are 0.2 mm in length.

The same cross-seeding stock from the optimized H2L6 crystals was used for the C836 complex crystallization by MMS. The screen, a subset of the original 192 ‘fast screen’ conditions, included 24 selected conditions from each of the Hampton PEG/Ion Screen and in-house PEG 8000 grid screens and was performed at 6 mg ml^−1^ protein concentration. Crystal formations of poor quality appeared in several drops in a range of conditions after 2 d (Figs. 3 ▶ *a* and 3 ▶ *b*). A number of drops remained clear. A mixture of these crystals obtained in different conditions produced a ‘self-seeding’ stock.

Both ‘cross’ and ‘self’ seeds were used in the MMS optimization, in which the protein concentration was 9 mg ml^−1^. To minimize the number of experiments, the optimization screens included only one buffer, 0.1 *M* HEPES buffer pH 7.5, and one of the four salts (sodium formate, sodium tartrate, ammonium citrate, lithium citrate) at 0.2 *M* concentration in the presence of 18–22% PEG 3350. X-ray-quality crystals were obtained in both the self-seeding and the cross-seeding experiments within 2 d (Figs. 3 ▶ *c*–3 ▶ *f*). It is worth noting that the cross-seeds yielded crystals in all four salts, whereas the self-seeds only gave crystals in ammonium citrate. The seed quality may be one reason for this difference.

The best cross-seeding conditions were 20% PEG 3350, 0.2 *M* sodium tartrate, 0.1 *M* HEPES pH 7.5 (Fig. 3 ▶ *f*). The crystals belonged to the monoclinic space group *P*2_1_, with unit-cell parameters *a* = 76.62, *b* = 65.56, *c* = 118.74 Å, β = 107.02°. The best self-seeding conditions were 18% PEG 3350, 0.2 *M* ammonium citrate, 0.1 *M* HEPES pH 7.5 (Fig. 3 ▶ *c*). These crystals were isomorphous to the cross-seeded crystals. Both types of crystals diffracted to 2 Å resolution.

## 4. Discussion

The initial crystallization screening for all three complexes included 192 conditions from the commercial and in-house screens. Despite sequence similarities between the complexes, only one experiment (28% PEG 8000, 0.1 *M* MES pH 6.5) with H2L6 produced needle-like crystals. Since a classical optimization of the conditions for improving these crystals proved fruitless, we faced a choice of either using these microcrystals as seeds or extending, perhaps significantly, the initial screening. The latter option would certainly require much more protein and would not after all guarantee the result. In contrast, the first option proved to be very efficient and given the variety of successful conditions appears to be more robust and reliable.

MMS using the initial H2L6 microcrystals was performed in 48 conditions of the standard Hampton PEG/Ion Screen and in eight additional conditions. Multiple hits amenable to optimization appeared overnight under a number of conditions that included 20% PEG 3350 and 0.2 *M* salt with a pH in the range 5.0–8.0. All drops contained a large number of crystals, indicating that the seed concentration was too high. Optimization was achieved by simply diluting the seed stock, resulting in X-ray-quality crystals from the same PEG/Ion Screen.

M1295 differs in sequence from H2L6 at only four positions, none of which are involved in lattice contacts in the H2L6 crystal form that was used for structure determination. Owing to this similarity, it was not surprising that the M1295 crystals were isomorphous to the H2L6 crystals. An interesting observation resulting from these experiments was that the X-­ray-quality crystals of M1295 were obtained from the same screen that was used unsuccessfully in the initial screening of this complex. MMS with H2L6 seeds yielded several crystals suitable for X-ray diffraction studies and a large number of hits that could be easily optimized by simple seed dilution.

The same MMS procedure was applied to the C836 complex. The amino-acid sequence of C836 differs significantly from that of the other two Fabs since C836 contains mouse variable domains. Despite the differences and the reduced size of the MMS screen (only 48 conditions), a number of hits were obtained that could be optimized. However, conditions that favored the growth of large crystals were not among the 48 conditions selected for the MMS screen. A different set of 12 ‘optimized’ conditions based primarily on the H2L6 results was used with the same seed stock as before and yielded X-ray-quality crystals. This experiment showed that although the MMS screen may be less extensive than the initial ‘nucleation’ screen, it still must contain a sufficient array of refined conditions to find crystal-growth conditions. From a practical perspective, to conserve protein and time we start with a limited set of conditions and then extend it, if necessary, through additional screens covering different pH, reagent concentrations or additives.

Besides the reservoir composition, the protein and seed concentrations are other important parameters that affect crystal growth. For instance, in the C836 trials some drops remained clear after several days at a protein concentration of 6 mg ml^−1^ but hits were identified within 24 h when the concentration was increased to 9 mg ml^−1^. In the refinement step, high protein concentrations in the drop may trigger additional nucleation or even precipitation and should be avoided.

In the current application of MMS, we do not have much control over the seed concentration. However, under conditions where showers of crystals appeared, dilution of the seed stock was carried out in order to reduce the number of crystals in each drop. This was a key factor in H2L6 crystallization optimization. Conversely, the seed concentration in the first round of C836 MMS was not high enough. The desired concentration was achieved by combining and mixing seeds from different conditions. The use of the frozen seed stock ensured constant seed concentration and reproducibility of the experiments. We did not notice any decrease in seed concentration after repeated freeze–thaw cycles as judged by the number of crystals in the drop.

The introduction of seeds into the crystallization droplet increased the hit rate in all three cases described in this report. The effect was particularly noticeable for M1295 and C836, for which no hits were obtained from the initial screening. After MMS, crystals of various quality were observed in 18 of 192 conditions for M1295 and in seven of 24 conditions for C836. The question remains whether the seeds themselves or the seed-stabilization solution cause the effect. In our experiments, the dilution of the seed stock with the same stabilization solution reproducibly decreased the number of crystals in the drops. This may be the strongest argument supporting the role of seeds in nucleation.

Although published results with MMS have described the self-seeding protocol (D’Arcy *et al.*, 2007 ▶; Baumgartner *et al.*, 2009 ▶) and cross-seeding with protein derivatives (Walter *et al.*, 2008 ▶), there may be no restriction to the composition of the cross-seeds used to promote crystal growth. The crystallization of the C836 complex is an example of successful cross-seeding with a different protein in a different crystal form.

**Figure 4:**
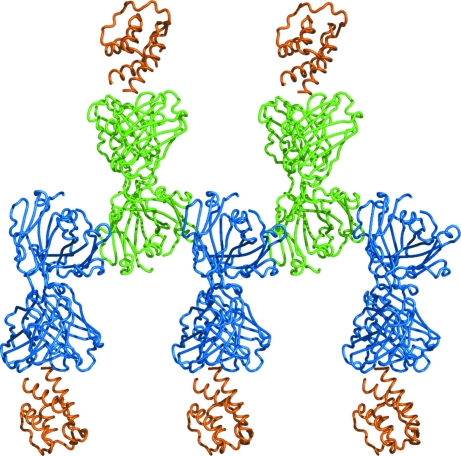
Common crystal contacts in the H2L6 complex and the C836 complex. Fabs in the same color (blue or green) are related by a crystallographic translation. Fabs in different colors are related by a crystallographic twofold axis. IL-13 is shown in orange.

H2L6 and C836 have identical constant domains but different variable domains. The H2L6 crystals are ortho­rhombic (space group *P*2_1_2_1_2_1_, unit-cell parameters *a* = 64, *b* = 73, *c* = 115 Å). The C836 crystals are monoclinic (space group *P*2_1_, unit-cell parameters *a* = 77, *b* = 66, *c* = 119 Å). Both forms have approximately the same packing density, with *V*_M_ values of 2.23 Å^3^ Da^−1^ for H2L6 and 2.35 Å^3^ Da^−1^ for C836. Analysis of intermolecular contacts in these crystals revealed one type of contact that is common to both crystal forms: a β-­bridge between the light and heavy chains of contacting Fabs. This interaction yields a row of Fab molecules linked through their constant domains (Fig. 4 ▶). Interactions involving the variable domains and IL-13 are quite different in the two crystal forms. It is possible that the interactions between the constant domains formed the basis of the crystal lattice that served as a nucleus for the cross-seeded crystal growth.

In conclusion, MMS proved to be a fast, easy and reliable method for refinement of crystallization conditions. Once the initial conditions have been established, the number of crystals in the drop may be controlled by dilution of the seed stock, which often is sufficient to obtain large crystals. In our experience, the MMS method promotes crystal nucleation and increases the hit rate, thus reducing the size of the initial crystallization screen and saving time and protein. In some cases, MMS produces crystal forms that differ from those of the seeds. Further experiments may determine whether a ‘universal’ seed stock can produce enough hits for a given protein or class of protein whose members have significant sequence homology, such as Fabs.
